# Values and physical activity among sports science students in France and China: a transcultural analysis

**DOI:** 10.3389/fpsyg.2023.1304019

**Published:** 2024-01-04

**Authors:** Yan Liang, Olivier Rascle, Paul H. P. Hanel, Jian Yang, Nicolas Souchon

**Affiliations:** ^1^Sino-French Joint Research Center of Sport Science, College of Physical Education and Health, East China Normal University, Shanghai, China; ^2^Department of Sports Sciences, VIPS2 laboratory, Rennes 2 University University of Rennes 2 – Upper Brittany, Rennes, France; ^3^Department of Psychology, University of Essex, Colchester, United Kingdom; ^4^College of Physical Education and Health, East China Normal University Université Paris Nanterre, Nanterre, France; ^5^UFR STAPS, LICAE laboratory, Université de Paris, Nanterre, France

**Keywords:** human values, physical activity, cross-cultural research, France, China

## Abstract

**Objective:**

The aim of this study was to analyze the relationships between values and physical activity in France (a Western European individualistic country) and in China (an East Asian collectivist country).

**Method:**

Six hundred and twenty-seven sport science students in France (*N* = 308, *M*_age_ = 18.99, *SD* = 1.64) and China (*N* = 319, *M*_age_ = 20.44, *SD* = 1.09) completed the International Physical Activity Questionnaire long version and the Portrait Values Questionnaire.

**Results:**

In both France and China, moderated regression analysis revealed that hedonism positively/negatively predicted physical activity, while security-societal, security-personal, and conformity-rules values negatively predicted physical activity. In contrast, stimulation and universalism-nature values positively predicted physical activity only in France. In China, benevolence and benevolence-care positively predicted physical activity, while power dominance negatively predicted physical activity. Additionally, we found evidence of measurement invariance of the value questionnaire.

**Discussion and conclusion:**

Our findings add to the literature by showing that the value–behavior link is partly the same across countries and partly different. Further, our findings show that for certain populations, the previously established hierarchy of human values does not replicate.

## Introduction

Values (e.g., freedom, pleasure) can be defined as abstract ideals that are important guiding principles in one’s life (Schwartz, 1992; Sagiv and Roccas, 2021). Values motivate people over time and across situations (Schwartz, 1992; Maio, 2010; Arieli et al., 2020) and represent a central part of identity and self-concept (Verplanken and Holland, 2002; Maio, 2016). As such, values shape both individuals’ current and future health-related behaviors. There is evidence that specific values relate to several everyday health-related behaviors such as smoking (Nieh et al., 2018), drinking alcohol (Inman et al., 2017; Rudnev and Vauclair, 2018), or drug-use (Sanchez et al., 2018; for an overview, see Hanel et al., 2022).

One important health-related behavior is engagement in physical activity. The latter can be defined as any bodily movement produced by skeletal muscles that requires energy expenditure and can be done at a variety of intensities and accumulated through work, domestic chores, transportation, or during leisure time, or when participating in sport, walking, cycling, active recreation, and active play (World Health Organization, 2022). Physical activity can be considered as a complex system of relationships affected by multiple levels of the surrounding environment, from immediate family and school settings to broad cultural values and customs (Lee and Park, 2021).

However, an understudied question is how values impact physical activities and whether this impact is invariant across cultures. Previous research suggests that the pattern might be different across countries. Culture can affect the specific behaviors that individuals spontaneously associate with a specific value (Hanel et al., 2017, 2018). We focus on human values as predictors, because they are the core of a culture (e.g., Sagiv and Schwartz, 2022) and they influence human behaviors (e.g., Maio, 2010; Sagiv and Roccas, 2021). Hence, it is conceivable that people attribute a different meaning to physical activities across countries and associate physical activity with different values across countries.

Focusing on sports science students in France and China, the aim of this study was consequently to establish which values predict physical activity in France (a West European individualistic country) and in China (an East Asians collectivist country), and whether these associations differ between countries.

### Schwartz’s model of values

**Figure 1:**
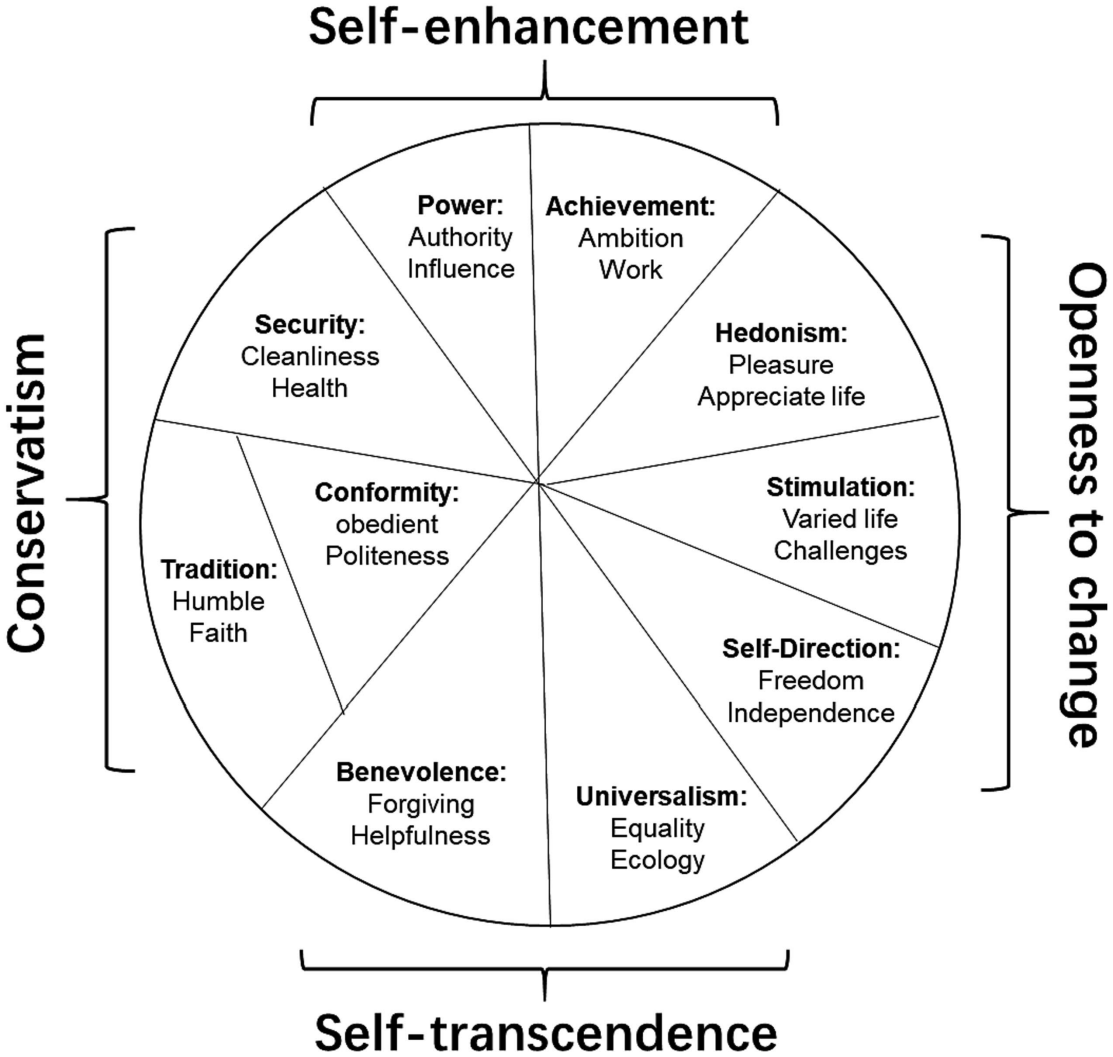
Schwartz’s (1992) circumplex model of personal values displaying 10 value types (bold font) and examples of values in each type (normal font).

Values are grounded in one or more of three universal requirements of human existence: the needs of individuals as biological organisms, requisites of coordinated social interaction, and survival and welfare needs of groups (Schwartz, 1992). Schwartz’s (1992) model of values is considered to be one of the most important theoretical advances in the field of human values (Maio, 2016). Schwartz’s (1992) model is central in psychology, but also in other disciplines such as philosophy, sociology, and anthropology (Maio, 2010). Its structure (Figure 1) has been supported in more than 100 countries (e.g., Schwartz et al., 2017; Sagiv and Schwartz, 2022).

Schwartz (1992) universally identified ten broader categories of values (see Supplementary Table S1) and organized these value types into a quasi-circumplex to highlight the dynamic relationships between them (Figure 1). Schwartz represents motivationally compatible values (e.g., stimulation and self-direction) adjacently in the model, while motivationally incompatible values are on opposite sides in the model (e.g., self-direction and security).

Moreover, the model (Figure 1) distinguishes between values conveying (1) openness to change: i.e., stimulation, self-direction, and hedonism; (2) conservation of the status quo: i.e., tradition, conformity, and security; (3) serving self-interests: i.e., power and achievement; and (4) transcendence of self-interests in the service of others: i.e., universalism and benevolence, all of which explain different variables such as behavior (e.g., Maio, 2016; Sagiv and Roccas, 2021).

More recently, Schwartz et al. (2012) proposed a refined model that divided the motivational quasi-circumplex from 10 into 19 value types. For example, security was divided into security-personal and security-societal, and conformity into conformity-interpersonal and conformity-rules. This was done to get a better understanding of the relations of values with other variables. For example, behavior that aimed to increase one’s personal safety was as expected connected to security-personal, but not connected with security-societal (Schwartz and Butenko, 2014).

Research has shown that values predict emotional reactions (e.g., Conte et al., 2023), political orientation (e.g., Caprara et al., 2006), prejudice (e.g., Wolf et al., 2019), well-being (e.g., Sortheix and Schwartz, 2017), and a series of health behaviors (Rudnev and Vauclair, 2018). However, only four studies to our knowledge have tested the relationships between values and physical activity using Schwartz’s (1992) model (Worsley et al., 2013; Souchon et al., 2015; Skimina et al., 2019, 2021).

### Values and physical activity

In one of these studies, the aim was to analyze relationships between values and more general health behaviors (Worsley et al., 2013), while in two other studies, the aim was to test relationships between values and more general typical everyday behaviors (Skimina et al., 2019, 2021). This particularity explains why the authors in these three studies used a short measure of self-reported frequency of everyday behavior rather than a well-validated self-reported measure of physical activity.

Skimina et al. (2021) found that security and security-personal values were negatively related to physical activity, while stimulation was positively related to physical activity. In another study, Skimina et al. (2019) found that both security-personal and conformity-interpersonal were negatively related to physical activity. Finally, measuring only universalism and conformity values, Worsley et al. (2013) found a positive relationship between universalism and physical activity.

To the best of our knowledge, only one study tested the relationships between values and a well-validated self-reported measure of physical activity. Souchon et al. (2015) investigated the associations between a value measure, the PVQ-40, and the Godin Leisure Time Exercise Questionnaire (GLTEQ, Godin and Shephard, 1985). Results indicated that stimulation, hedonism, and achievement were positively related to physical activity, while tradition was negatively related to physical activity. These findings were consistent with Skimina et al.’s (2021) results on the role of stimulation values, but were less reliable for other values.

Moreover, none of the studies explored whether human values predict physical activity in China. Nevertheless, the relationships between values and behaviors are complex and research has shown that individuals in different cultures prototypically instantiate different behaviors to the same values. For example, security in Brazil is related to avoiding gunshots when walking in the street, while in United Kingdom, security is related to being able to finance higher education for children (Hanel et al., 2018; Coelho et al., 2022).

### The present study

Our aim in this study was to establish if values predict physical activity in France and China in the same way or differently. The comparison between French participants and Chinese participants is particularly relevant as there is a remarkable social cognitive distinction in cultures. Western culture, particularly those influenced by European traditions, tend to emphasize individual freedom, autonomy, and personal achievement. In contrast, Eastern cultures, such as those found in Asia, place greater emphasis on collective harmony, familial ties, and societal obligations (Vignoles et al., 2016). A large amount of cross-culture studies have focused on the comparison across Western and Eastern cultures to better understand the similarities and diversity across two culture paradigms and foster a deeper understanding and appreciation of the global community (e.g., Honka et al., 2019; Ho et al., 2022). We expect the present study could reveal the similarities and/or diversity in the relationships between values and physical activity to provide further valuable suggestion to the physical activity promotion plans in China and France (World Health Organization, 2018).

For the participants, we focused on sports science students in France and China. The aim of sports science students is not to become professional athletes but to work in the general management of physical and sporting activities (e.g., teachers or working in health domain). This population has not been studied in research on values–physical activity relationships before. Sport science students have presumably a positive attitude toward physical activity and should often engage in it. Given that a high level of physical activity is desirable because it is associated with physical and mental health benefits, investigating predictors of physical activity in a population that routinely engage in it can have important policy implications.

### Hypotheses in the present study

#### Openness to change

Openness to change values emphasize independence of thought, action, and feelings, and readiness for change (hedonism, self-direction, and stimulation). Souchon et al. (2015) and Skimina et al. (2021) found that hedonism (pleasure) and stimulation (excitement, novelty, and challenge in life) were positively associated with physical activity. These results suggest that pleasure, emotion, and challenges experienced during physical activity could be an important drive for people who value hedonism or stimulation. We expect to replicate those findings in France with sport science students.

However, compared to Western culture, the cultural tradition in China inclines to Daoism, which advocates the pursuit of a peaceful life rather than a stimulating one (no excess). Daoism emphasizes respect for life, nature, adaptation to changes, supplemented by appropriate moderate exercise (Li, 2015). From the perspective of Daoism, we propose that stimulation does not significantly relate to physical activity in China. Finally, self-direction has shown a non-significant relationship to physical activity in previous studies (Souchon et al., 2015; Skimina et al., 2021). We expect no relationship between self-direction and physical activity in this study.

*H1*: Hedonism and stimulation are positively associated with physical activity in France but not in China.

*H2*: Self-direction is not significantly associated with physical activity in France and China.

#### Self-transcendence

Self-transcendence values emphasize concern for the welfare and interests of others (universalism and benevolence) in Schwartz’s (1992) model. In the physical activity domain, Worsley et al. (2013) found in Australia a positive relationship between universalism and physical activity, but Souchon et al. (2015) in France and Skimina et al. (2019, 2021) in Polona did not find any associations. Consequently, we do not have clear expectations on the role of universalism in France.

In China, we speculate that an association between universalism and physical activity might exist, because one of the most important doctrines of Chinese thoughts, Confucianism, emphasizes Ren (仁), which is very similar to benevolence in Schwartz’s (1992) model and which emphasizes the need to develop virtuous qualities through proper social engagement, such as to love parents, siblings, and the whole family, but also to share activities with others (Low, 2011; Ahmad et al., 2020). We therefore hypothesize that benevolence values are positively associated with physical activity, because participants are eager to integrate into society and involve more prosocial behavior.

*H3*: Universalism and benevolence values are positively associated with physical activity in China.

#### Conservation values

Conservation emphasizes order, self-restriction, preservation of the past, and resistance to change (conformity, tradition, and security). In previous studies, conservation values were negatively related to physical activity. Especially, security-personal (Skimina et al., 2019), conformity-interpersonal (Skimina et al., 2021), and tradition were negatively related to physical activity (Souchon et al., 2015). Consequently, conservation values should be negatively related to physical activity in this research both in France and China.

This reasoning is further supported by Confucianism, which perceives the body as the medium of moral practice. Conformity and tradition are correlated with body ideology in tradition Daoism. Daoism emphasizes respect for life and adaptability to changes, supplemented by appropriate moderate exercise (Li, 2015). One of the functions of ritual training in body is to maintain the order of the superior and inferior in social order (Guan, 2022). Zuo and Wang proposed that the anchoring concepts “psyche is precious rather than body” and “advocate morality development rather than physical strength” have certain limitations and have even played a negative role in the development of Chinese sports (Zuo and Wang, 1998). Therefore, we hypothesize that the conservation values conformity, tradition, and security are negatively associated with physical activity in China.

*H4*: Conservation values (including humility and face) are negatively related to physical activity both in France and China.

#### Self-enhancement

Self-enhancement is defined as the pursuit of one’s own interests and relative success and dominance over others (achievement and power; Schwartz, 1992). Achievement is defined as personal success through demonstrating competence according to social standards. As the sporting context is an achievement area (Gröpel et al., 2016), achievement values may be related to a higher level of physical activity especially among sport science students because they tend to be passionate about sport and physical activity. This prediction is in line with previous research showing this within the general population in France (Souchon et al., 2015). Consequently, we expect that in France, achievement and physical activity are positively associated. However, since we are assuming that in China, benevolence values, which are opposed to achievement values in Schwartz’s (1992) model, are positively associated with physical activity due to the cultural importance of Ren values, the association between achievement and benevolence will be non-significant.

Moreover, power value is defined as social status and prestige, control or dominance over people and resources. Although power values are motivationally compatible with achievement values which might positively predict physical activity, power values were found to be unrelated with physical activity in France (Souchon et al., 2015). Consequently, we expect no relationship between power and physical activity in France.

Finally, traditional Chinese culture emphasizes “self-cultivation, family management, state governance, and bringing peace to all under heaven” as the four terminal goals in life (Zhang, 2023). Self-cultivation is explained as the cultivation of mind, which advocates improving one’s mental character through studying knowledge rather than body training (Li, 2015). Therefore, studying to pass the imperial examination was basically the only approach to enter the higher societal class in China (Ko, 2017), whereas excessive physical labor was regarded as a sign of the lower class in Chinese society (Lu, 2012). Hence, we postulate that Chinese participants who attach more importance to power tend to engage in less physical activity due to the idea that “psyche is more precious rather than body.”

*H5*: Achievement is positively associated with physical activity in France, but not in China.

*H6*: Power is negatively associated with physical activity in China, but not in France.

The hypotheses are summarized in Table 1.

**Table 1:** Hypothesis synthesis.

	*H1*: Hedonism and stimulation are positively associated with physical activity in France, but not in China *H2*: Self-direction does not significantly predict physical activity in France and China.
Openness to change	
Self-transcendence	*H3*: Universalism and benevolence are positively associated with physical activity in China.
Conservation	*H4*: Conservation values (including humility and face) are negatively related to physical activity both in France and in China
Self-enhancement	*H5*: Achievement is positively associated with physical activity in France, but not in China. *H6*: Power is negatively associated with physical activity in China, but not in France.

## Method

### Participants and procedure

A power analysis using G*Power 3.1.9.7 (Faul et al., 2009) revealed that to detect a small effect size of *f* = 0.15 with a power of 0.95 and α = 0.05 in a 2 × 2 × 2 between-subjects design, a total sample size of 580 is required. While previous research (e.g., Worsley et al., 2013; Skimina et al., 2019) found small and medium effect sizes, we assumed a small effect size to be conservative. Six hundred and twenty-seven sport science students completed the study individually in France and in China. The study included two hundred and eleven women and four hundred and fourteen men in France and China (see Supplementary material for more information). Table 2 describes the sample in France and China.

**Table 2:** Description of the sample.

	Sum (*N* = 627)		France (*N* = 308)		China (*N* = 319)	
	Male	Female	Male	Female	Male	Female
*N*	414	211	196	112	220	99
Age (SD)	19.88 (0.60)	19.73 (1.57)	19.05 (1.64)	18.89 (1.65)	20.61 (1.14)	20.07 (0.88)
BMI (SD)	22.35 (2.38)	21.95 (2.38)	22.13 (2.39)	21.65 (2.38)	22.55 (2.16)	20.61 (2.25)

Participants provided informed consent prior to completing the pen-and-paper questionnaire in Chinese or French in class before lectures in a sport science department. Participants were informed that participation was voluntarily and they were able to drop out at any point. All procedures performed in our studies were in accordance with the ethical standards of the 1964 Helsinki declaration and its later amendments, and also in line with the American Psychological Association (2017) and the British Psychological Society (2009). Moreover, the present study received ethical approval by the University Committee on Human Research Protection of East China Normal University (HR 725–2021). The data can be found online on https://osf.io/kjz5d/?view_only=c9fef240a011443abaad89a235023483.

### Measures

#### Values

In France and in China, participants first completed the PVQ-RR-57 (Portrait Values Questionnaire refined model; Schwartz et al., 2017), which we downloaded from the Schwartz’s repository of value instruments^1^, the official website of the Schwartz Value Questionnaire. The PVQ-RR-57 has been validated both in France and in China (Schwartz and Cieciuch, 2022). It measures each of the 19 value types with three items. Each question refers to a portrait of an individual that participants are asked to identify with by responding from 1 (not at all like me) to 6 (very much like me). For example, the items that measure the importance male participants attach to self-direction-thought are: “It is important to him to form his views independently,” “It is important to him to develop his own opinions,” and “It is important to him to figure things out himself.” The 57 items can be combined into four higher order values, 10 basic values, or 19 narrowly refined values.

#### Self-reported physical activity behavior

To measure physical activity, we used the well-validated International Physical Activity Questionnaire long version (IPAQ-L) which has been successfully used cross-culturally (Bauman et al., 2009) and also been validated in French by Crinière et al. (2011) and in Chinese by Fan et al. (2014).

The IPAQ-Long version contains 27 items and examines four PA domains (job-related, transportation, domestic, and leisure). The results can be described as a continuous score, by domain, and by intensity of PA (moderate or vigorous). PA is found according to the following formula (in MET-min-week): the number of days making physical activity X mean duration of the activity per day X the energy cost of the activity. The energy cost of an activity is expressed in METs (the metabolic equivalent of task).

MET is the ratio of the energy expenditure during a given activity divided by the resting energy expenditure. The following MET values were drawn from the scoring protocol: 3.3 for walking, 4 for moderate intensity PA, 8.0 for vigorous PA, 6.0 for cycling, 5.5 for vigorous PA in a garden or yard, and 3.0 for domestic activities (IPAQ group, 2005).

### Data cleaning and analysis

Following best practice recommendations, participants who responded more than 45 times with the same response option or had more than 28 missing values were excluded (Schwartz, 2021). Twelve French participants and 13 Chinese participants were excluded, leaving 308 French participants and 319 Chinese participants. Analyses were conducted using STATISTICA 13.0 and SPSS 24.0 software IBM Corp (2016). IBM SPSS Statistics for Windows, Version 24.0. Armonk, New York: IBM Corp.

To test for configural, metric, and scaler invariance of the PVQ-RR in our sample, we used the simultaneous factor analysis framework (Schwartz et al., 2012, see analysis in Supplementary Material).

#### Reliability

We used Cronbach’s alpha to report the internal reliability for the 19 narrowly defined values, 10 broader values, and four higher order values in France and in China. Since some of the 19 value types are broad, some Cronbach’s alphas were below 0.60 (Graham et al., 2011). This is in line with the literature (e.g., Schwartz et al., 2012). In contrast, the internal reliabilities of all four higher order value types were satisfactory. They ranged from 0.75 to 0.84 in France and 0.76 to 0.87 in China (see the confirmatory factor analysis, measurement invariance, and fit of the 19 values to the circular structure in the Supplementary Material).

#### Other statistical analysis

We analyzed global physical activity and mean values with mixed ANOVAs and we analyzed the relationships between values and global physical activity with moderated regression analysis (see in Supplementary Tables S8–S13 the interrelations between four values, 10 values, 19 values and in Supplementary Table S14, the interrelations between values and physical activity in the four specific domains). Specifically, we tested the relationships between values and physical activity using one single value type at a time. Following Schwartz’s recommendations, we used values centered on a person level within the ANOVA and within the regressions.

Moreover, we tested in simple regressions whether gender, *β* = 0.08, *p* < 0.05 (women −0.5 and men +0.5), age, *β* = −0.10, *p* < 0.01, BMI (weight: kg /height: meters^2^), *β* = 0.06, *p* = 0.141, country (France +0.5 and China −0.5), *β* = 0.16, *p* < 0.001, and competition level (coded 0 “leisure time,” 1 “low local level,” 2 “high local level,” “3 low intermediate level,” 4 “high intermediate level,” “5 low national level,” 6 “high national level” and “7 international level”), *β* = 0.28, *p* < 0.001, predicted global physical activity. As the results were significant for gender, age, country, and competition level, we tested in multiple regressions whether the effects obtained in the main analyses remained significant after controlling for the impact of these variables.

## Results

### Descriptive statistics

Global physical activity was analyzed with a 2 × 2 × 2 between-subjects ANOVA, with country (France vs. China), gender (men vs. women), and competition statement (competition vs. leisure) as factors. The adjusted *R*^2^ was 0.096, and the model was significant, *F* (7,619) = 10.50, *p* < 0.001.

The results indicated a marginally significant main effect of country, *F* (1,619) = 3.41, *p* = 0.065, *ŋ*^2^ = 0.005, a significant main effect of gender, *F* (1,619) = 4.87, *p* = 0.027, *ŋ*^2^ = 0.027, a significant main effect of competition, *F* (1,619) = 32.08, *p* < 0.001, *ŋ*^2^ = 0.049, and a significant two-way interaction between country and competition, *F* (1,619) = 11.71, *p* < 0.001, *ŋ*^2^ = 0.018.

The marginally significant main effect of country suggested that participants in France (*M_France_* = 9432.21, *SD* = 6311.27) were slightly more physically active than participants in China (*M_China_* = 7312.88, *SD* = 6600.96). The significant main effect of gender revealed that men (*M_men_* = 8749.57, *SD =* 7024.16) were more physically active than women (*M_women_* = 7573.96, *SD* = 5398.10). The significant main effect of competition indicated that participants who took part in competition (*M_men_* = 10210.63, *SD =* 7071.50) were more physically active than participants who did not take part in competition (*M_men_* = 6661.44, *SD =* 5506.81). The interaction between country and competition showed that participants who took part in competition in France (*M* = 10027.32, *SD =* 6765.11) and in China (*M* = 10569.98, *SD =* 7659.04) had a similar level of physical activity*, p = 0.*465, while participants who did not take part in competition were more physically active in France (*M* = 8361.03, *SD =* 5260.28) than in China (*M* = 5803.86, *SD =* 5440.12*, p* < 0.001).

### Regression analysis

#### Higher order values and physical activity in France and in China.

Supplementary Table S5 describes the regression analysis made for testing whether higher order values, controlling for the influence of gender (coded −0.05 for female and + 0.05 for male), age, country (coded a − 0.05 for France and + 0.05 for China), and competition level (from 0 “leisure” to 7 “international competition level”), predict global physical activity.

The results indicate that only openness to change values predicted global physical activity across the two samples from France and China (*B* = 0.12, *p* = 0.002). This effect remained significant even after controlling for the influence of gender, *B* = 0.10, *p* = 0.009, age, *B* = 0.00, *p* = 0.992, country, *B* = 0.04, *p* = 0.305, and competition level, *B* = 0.26, *p* < 0.001, *R*^2^ = 0.106, *F* (5,621) = 15.87, *p* < 0.001.

### Ten broader categories of values and physical activity in France and in China

Tables 3–5 present significant regression analyses (see the non-significant regression analysis in Supplementary Tables S6, S7) testing how the single-value categories predict global physical activity, controlling for the influence of gender, BMI, age, country, and competition level.

**Table 3:** Results of moderated regressions for hedonism, stimulation, and self-direction values with country as the moderator in predicting global physical activity.

		Global physical activity		
		T	*p*	*B*
Hedonism	*R*^2^ = 0.047, *F* (3,623) = 11.29, *p* < 0.001				Hedonism	1.94	0.052	0.08	Country	2.91	0.003	0.12	Hedonism X country	0.94	0.346	0.03	*R*^2^ = 0.097, *F* (5,621) = 14.55, *p* < 0.001				Gender	2.58	0.010	0.10	Age	0.16	0.871	0.007	Country	−1.44	0.148	0.06	Competition level	6.57	<0.001	0.26	Hedonism	1.79	0.073	0.07
Stimulation	*R*^2^ = 0.047, *F* (3,623) = 11.29, *p* < 0.001				Stimulation	3.32	0.006	0.11	Country	2.75	<0.001	0.13	Stimulation X country	2.62	0.008	0.10
France	*R*^2^ = 0.123, *F* (4,303) = 11.81, *p* < 0.001				Gender	2.53	0.011	0.13	Age	1.55	0.121	0.08	Competition level	4.50	<0.001	0.24	Stimulation	4.86	<0.001	0.26
China	*R*^2^ = 0.099, *F* (4,314) = 9.75, *p* < 0.001				Gender	2.02	0.043	0.11	Age	−1.98	0.047	−0.11	Competition level	5.38	<0.001	0.29	Stimulation	0.51	0.611	0.03
Self-direction-action	*R*^2^ = 0.028, *F* (3,623) = 7.13, *p* < 0.001				Self-direction-action	−0.19	0.841	−0.01	Country	4.11	<0.001	0.16	Self-direction-action X country	−2.03	0.042	−0.08
France *R*^2^ = 0.061, *F* (4,303) = 6.03, *p* < 0.001				
	Gender	2.09	0.036	0.11	Age	1.50	0.133	0.08	Competition level	3.89	<0.001	0.22	Self-direction-action	−1.44	0.149	−0.08
China *R*^2^ = 0.099, *F* (4,314) = 9.77, *p* < 0.001				
	Gender	2.00	0.046	0.11	Age	−1.96	0.050	−0.11	Competition level	5.25	<0.001	0.28	Self-direction-action	0.55	0.580	0.03

Gender: −0.05 for women and + 0.05 for men, Country: −0.05 for France and + 0.05 for China.

**Table 4:** Results of moderated regressions for universalism-nature, benevolence, and benevolence-care values with country as the moderator in predicting global physical activity.

		Global physical activity		
		*T*	*p*	*B*
Universalism-nature	*R*^2^ = 0.030, *F* (3,623) = 7.62, *p* < 0.001				Universalism-nature	−0.15	0.875	0.00	Country	4.11	<0.001	0.16	Universalism-nature X country	2.40	0.016	0.10
France	*R*^2^ = 0.074, *F* (4,303) = 7.20, *p* < 0.001				Gender	2.24	0.025	0.12	Age	1.56	0.118	0.08	Competition level	4.37	<0.001	0.25	Universalism-nature	2.53	0.011	0.14
China	*R*^2^ = 0.100, *F* (4,314) = 9.85, *p* < 0.001				Gender	1.76	0.079	0.10	Age	−1.89	0.058	−0.10	Competition level	5.28	<0.001	0.29	Universalism-nature	−0.77	0.438	−0.04
Benevolence	*R*^2^ = 0.038, *F* (3,623) = 9.35, *p* < 0.001				Benevolence	0.88	0.375	0.04	Country	3.27	0.001	0.04	Benevolence X country	−3.25	0.001	−0.13
France	*R*^2^ = 0.051, *F* (4,291) = 4.98, *p* < 0.001				Gender	1.85	0.064	0.10	Age	1.14	0.255	0.06	Competition level	3.77	<0.001	0.22	Benevolence	−1.10	0.269	−0.06
China	*R*^2^ = 0.109, *F* (4,314) = 10.76, *p* < 0.001				Gender	2.12	0.034	0.11	Age	−1.85	0.064	−0.10	Competition level	5.02	<0.001	0.27	Benevolence	1.96	0.050	0.11
*Benevolence-care*	*R*^2^ = 0.033, *F* (3,623) = 8.14, *p* < 0.001				Benevolence-care	1.10	0.269	0.04	Country	3.74	<0.001	0.15	Benevolence-care X country	−2.61	0.009	−0.10
France	*R*^2^ = 0.058, *F* (4,303) = 5.73, *p* < 0.001				Gender	2.05	0.040	0.11	Age	1.28	0.198	0.07	Competition level	4.05	<0.001	0.23	Benevolence-care	−0.99	0.322	−0.05
China	*R*^2^ = 0.107, *F* (4,314) = 10.55, *p* < 0.001				Gender	2.10	0.036	0.11	Age	−1.83	0.067	−0.10	Competition level	5.14	<0.001	0.28	Benevolence care	1.76	0.078	0.09

Gender: −0.05 for women and + 0.05 for men, Country: −0.05 for France and + 0.05 for China.

**Table 5:** Results of moderated regressions for conformity-rules, security, security-personal, and power-dominance values with country as the moderator in predicting global physical activity.

		Global physical activity		
		*T*	*p*	*B*
Conformity-rules	*R*^2^ = 0.029, *F* (3,623) = 7.41, *p* < 0.001				Conformity-rules	−1.74	0.080	−0.07	Country	3.19	0.001	0.13	Conformity-rules X country	−0.81	0.414	−0.03	*R*^2^ = 0.100, *F* (5,621) = 14.97, *p* < 0.001				Gender	2.43	0.015	0.09	Age	−0.04	0.967	−0.01	Country	1.33	0.181	0.06	Competition level	6.68	<0.001	0.27	Conformity-rules	−2.25	0.024	−0.09
Security	*R*^2^ = 0.033, *F* (3,623) = 8.26, *p* < 0.001				Security	−1.75	0.078	−0.07	Country	3.49	<0.001	0.18	Security X country	−1.33	0.180	−0.07	*R*^2^ = 0.100, *F* (5,621) = 14.92, *p* < 0.001				Gender	2.33	0.019	0.09	Age	−0.03	0.975	−0.01	Country	−1.64	0.101	0.07	Competition level	6.59	<0.001	0.26	Security	−2.21	0.027	−0.09
Security-personal	*R*^2^ = 0.043, *F* (3,623) = 10.57, *p* < 0.001				Security-personal	−1.62	0.103	−0.06	Country	3.79	<0.001	0.15	Security-personal X country	−2.83	0.004	−0.11
France	*R*^2^ = 0.089, *F* (4,303) = 8.49, *p* < 0.001				Gender	1.46	0.144	0.08	Age	1.17	0.242	0.06	Competition level	3.96	<0.001	0.22	Security-personal	−3.35	<0.001	−0.19
China	*R*^2^ = 0.099, *F* (4,314) = 9.74, *p* < 0.001				Gender	2.05	0.041	0.11	Age	−1.91	0.056	−0.10	Competition level	5.33	<0.001	0.29	Security-personal	0.46	0.639	0.025
Power-dominance	*R*^2^ = 0.038, *F* (3,623) = 9.41, *p* < 0.001				Power-dominance	−1.52	0.128	−0.06	Country	3.10	0.001	0.13	Power-dominance X country	3.16	0.001	0.12
France	*R*^2^ = 0.055, *F* (4,303) = 5.52, *p* < 0.001				Gender	2.04	0.041	0.11	Age	1.29	0.194	0.07	Competition level	3.97	<0.001	0.23	Power-dominance	0.45	0.648	0.02
China	*R*^2^ = 0.125, *F* (4,314) = 12.40, *p* < 0.001				Gender	2.47	0.013	0.13	Age	−1.92	0.055	−0.10	Competition level	5.29	<0.001	0.28	Power-dominance	−3.11	0.002	−0.16

Gender: −0.05 for women and + 0.05 for men, Country: −0.05 for France and + 0.05 for China.

Specifically, there was a simple effect of hedonism values. This effect remained significant after controlling for gender, age, country, and competition level (Table 3). There was a significant interaction between stimulation values and country in the prediction of global physical activity (Table 3): stimulation values positively predicted global physical activity in France, but not in China, after controlling for gender, age, and competition level. There was also a significant interaction for self-direction action in predicting global physical activity. Nevertheless, the self-direction action values did not remain significant both in France and in China after controlling for age, gender, and competition level.

Moreover, there was a significant interaction between univer-salism nature and country in the prediction of global physical activity (Table 4). After controlling in France and in China for the influence of gender, age, and competition level, univer-salism nature positively predicted global physical activity in France but not in China. Also, there was a significant interaction between benevolence values and country in predicting global physical activity (see Table 4). Benevolence values positively predicted global physical activity in China but not in France after controlling for gender, age, and competition level. Specifically, there was a significant interaction between benevolence care and country. After controlling for gender, age, and competition level, benevolence care positively predicted global physical activity in China but not in France, albeit the effect was only marginally significant.

Finally, Table 5 shows significant simple effects and/or significant interaction effects for conformity-rules, security, security personal, and power-dominance. Conformity-rules and security negatively predicted global physical activity across countries after controlling for gender, age, country, and competition level. Also, there was a significant interaction between security personal values and country. Security personal negatively predicted global physical activity after controlling for gender, age, and competition level in France, but not in China. There was a significant interaction term between power dominance and country for global physical activity. Power dominance negatively predicted global physical activity in China, but not in France, after controlling for gender, age, and competition level. Additional analyses, such as correlations between values and physical activity separately for each of the four domain and country, are reported in the Supplemental materials (e.g., Supplementary Table S13).

## Discussion

Using sports science students as participants, our aim of this study was to establish if values predict physical activity in France and in China in the same way or differently. Our research adds to the literature because only very few studies have been undertaken on the relationships between values and physical activity previously (Worsley et al., 2013; Souchon et al., 2015; Skimina et al., 2019, 2021). All of these studies were conducted in Western countries and no study to our knowledge has been undertaken on the relationships between values and physical activity in China. Below, we discuss the findings in detail, as well as limitations and future directions.

### Openness to change and physical activity

Our first hypothesis was that hedonism and stimulation are positively associated with physical activity in France, but not in China. Our results tended to validate this hypothesis as hedonism predicted marginally positively a greater level of physical activity across countries. Also, stimulation positively predicted a greater level of physical activity in France, but not in China. The relationships between hedonism and stimulation and physical activity in France were overall consistent with our expectations and previous research (Souchon et al., 2015; Skimina et al., 2019, 2021).

These results showing that hedonism and stimulation are related to physical activity in France add to the literature showing that openness to change values predict antisocial and violent behaviors (e.g., delinquency) in real life (e.g., Benish-Weisman, 2015) or aggressive behaviors in sporting activities (e.g., Danioni and Barni, 2017). They also add to the literature showing that hedonism and stimulation are positively related to drinking behaviors (e.g., Rudnev and Vauclair, 2018) or internet consumption (Metin-Orta and Demirtepe-Saygili, 2021).

Interestingly, aggressive-antisocial behaviors, drinking behaviors, or internet consumption behaviors may all have, like physical activity, an addictive component (e.g., Rendi et al., 2007; Symons Downs et al., 2019). Someone may become dependent on physical activity as being dependent on being aggressive towards other people (e.g., fighting in a pub) due to dependency on physiological responses (e.g., searching for adrenaline or endorphin, Howard, 2011). An interesting question is why stimulation–hedonism did not relate to physical activity the same way in China.

Results have shown that a greater attachment to hedonism is marginally related to a greater level of physical activity across countries, but stimulation values are not related to physical activity in China. So, globally, hedonism–stimulation values are not related to physical activity in China. An explanation may be the importance attributed to Daoism in China. This cultural “conservation” principle emphasizes the importance of being in harmony with others, and reducing excessive exercise or avoiding aggressive body contact with others is the appropriate approach to practicing moral development in Confucianism (Li, 2015; Guan, 2022). Interestingly, our results indicated (see Supplementary Material) that French participants attached a higher importance to openness to change values than Chinese participants, while these latter attached more importance to conservation values, which is in line overall with previous research (e.g., Schwartz, 2006). Also, in the French sample hedonism values were the most important values, while security values were the most important values in the Chinese sample, albeit hedonism remained fairly important in China as well. This adds to the literature by showing that for young people who clearly like physical activity, the value hierarchy is somewhat different. Across 54 nations, Schwartz and Bardi (2001) found that for students, benevolence was most important, while hedonism ranked only in position 7 (out of 10). This suggests that there are some self-selection effects or socialization effects for sports science students (Bardi et al., 2014).

The simple fact that Chinese participants attached less importance to openness to change values does not explain intrinsically why stimulation values were not related to physical activity in China. A possibility is that physical activity in China would not be represented in memory as something fun, pleasant, and stimulating as it would in France. For example, Chinese individuals are the best players in table tennis in the world, but their very well-known method involves very hard work from a very young age (e.g., 8 h of training a day). The large number of players available all over the country presumably forces them to place less emphasis on the game’s playful dimensions and instead focus on the competitive side (e.g., authoritarian coaching, strong conformity to the rules, etc.); while in Europe, the hedonist–playful dimension of the game is seen as very important for motivating players over the long term (Lenartowicz, 2023). We therefore speculate that sport or physical activity is prototypically associated in memory in Europe with hedonism and stimulation, but not necessarily in China (Hanel et al., 2017, 2018). Previous research supports the notion that certain physical activities are differently perceived across countries. For example, cycling is perceived as tiresome and socially undesirable in China but as normative and even fashionable in Denmark (Bauman et al., 2009).

Our second hypothesis was that self-direction is not associated with physical activity in France and China. Our results validate this hypothesis as self-direction, self-direction action and thought, are not related to physical activity. This result is consistent with previous studies on the relationships between values and physical activity (e.g., Souchon et al., 2015). Interestingly, research has shown that attaching a greater importance to self-direction is related to a higher level of well-being (Sortheix and Schwartz, 2017). Also, people attaching greater important to self-direction and self-direction thought tend to be more academically successful (Vecchione and Schwartz, 2022). Nevertheless, self-direction is not related to physical activity. This is surprising as people attaching a greater xxx importance to self-direction and especially self-direction thought could better understand the importance of doing physical activity for their health or well-being.

### Self-transcendence value and physical activity

Our third hypothesis was that universalism and benevolence are positively associated with physical activity in China. Our results do not support this hypothesis. Indeed, a greater attachment to benevolence values was associated with a higher level of physical activity in China, while universalism values were not related to physical activity in this country. Moreover, a greater attachment to univer-salism nature predicted a greater level of physical activity in France. These results are nonetheless very interesting.

Ren principles in traditional Chinese culture, very close to benevolence values, promote sharing activities with others and then acting collectively in opposition to individualistically. Moreover, in China, due to a rapid population expansion, doing physical activity is becoming increasingly difficult, leading individuals to share it (e.g., large Qi Gong event) more and more collectively (Day, 2016). This may explain why, in China, benevolence values could be prototypically associated in memory with physical activity (Hanel et al., 2017, 2018). Sharing physical activity with other would be part of the culture in China.

In France, Souchon et al. (2015) as well as Skimina et al. (2019, 2021) did not find any relationship between universalism and physical activity within their samples. Nevertheless, the relationship between universalism nature and physical activity in our sample is in agreement with a previous study undertaken in Australia in which the authors found that a greater attachment to universalism predicted a higher level of physical activity habits (Worsley et al., 2013). Universalism nature values are defined as preservation of the natural environment (Schwartz et al., 2012) and people who attach a high importance to universalism values may like being outside (e.g., de Groot and Steg, 2007). Universalism nature-oriented people may be motivated to run, ride, or walk outside to be part of nature or at least because they need to be outside.

### Conservation value and physical activity

Our fourth hypothesis was that conservation values (including humility and face) are negatively related to physical activity both in France and in China. Our results tended to validate this hypothesis, as a greater attachment to conformity rules and security predicted a lower level of physical activity. Nevertheless, tradition, humility, and face were not related to physical activity. Also, a greater attachment to security personal predicted a lower level of physical activity in France, but not in China. These results are interesting and in agreement with previous studies undertaken on the relationships between values and physical activity (e.g., Souchon et al., 2015; Skimina et al., 2019).

Interestingly, at first glance, it appears surprising that security-personal negatively predicted physical activity, as health is part of security-personal values (Schwartz, 1992; Schwartz et al., 2012). Nevertheless, security-personal values are defined as the “Security of self and one’s immediate environment” (Schwartz et al., 2012) and doing physical activity implies taking some risks. Cycling as a means of transport may be perceived to be dangerous, especially in big cities. Also, security value-oriented people may be afraid to walk, run, or ride in some areas of big cities (e.g., because they might fear to being hit by a car; Foster and Giles-Corti, 2008). Security-oriented people could also be reluctant to meet other people to do physical activity (fear of meeting new people) and may feel easily threatened.

Security personal values are adjacent in Schwartz et al.’s (2012) model to conformity-rules values, which are defined as “Compliance with rules, laws, and formal obligations.” Our research also revealed that conformity-rules predicted a lower level of physical activity. In some circumstances, physical activity could violate conformity rules values. For example, the way people dress when running (or their nudity level) could be perceived to be socially inappropriate. Cycling in towns may also be perceived as inappropriate as it may be a problem for cars and road circulation in general. Understanding these processes may help politicians to better communicate with people who are valuing security to encourage them to be physically active (e.g., by emphasizing measures to be taken to improve safety for cyclists, if applicable).

Security-personal and conformity-rules values are “self-protection anxiety control values” with a social focus. They are directly in opposition in the Schwartz’s model with stimulation–hedonism values which are “growth, self-expansion, and anxiety free” values with a personal focus. Schwartz et al. (2017) demonstrated that value-expressive behaviors, such as physical activity in our present research, are regularly not the product of a single value (e.g., stimulation). Rather, values on one side of the motivational structure (here stimulation and hedonism) promote them whereas values on the opposite side of the motivational structure prevent them (here conformity rules and security personal). Thus, behaviors are a product of value trade-offs. Finally, this is particularly interesting that conservation values were related to physical activity the same way in both France and China.

### Self-enhancement value and physical activity

Our fifth hypothesis was that achievement is positively associated with physical activity in France, but not in China. Our results invalidated this hypothesis in France. Achievement was not related to physical activity both in France and China. In France, a possible explanation may be related to the diversity of values pursued by sport science students. Indeed, sport science departments in France, but also in China, are not devoted to developing professional sportsmen or sportswomen. Students most often study to become teachers in national education institutions or health institutes like hospitals (e.g., teaching physical activity (PA) to patients with obesity). For example, in our French sport science sample, the most important values were hedonism and benevolence (see Supplementary Material). Consequently, it was not so surprising to find no relationship in our sample between achievement values and physical activity. Students could not strongly associate in memory physical activity (PA) with achievement values in a sport science department (e.g., Hanel et al., 2018) or think about achievement values (i.e., value states) when they did physical activity in a sport science department (Skimina et al., 2019). They may simply relate achievement values to simple success in academia (Vecchione and Schwartz, 2022). Also, a statistical analysis (see Supplementary Table S8) indicates that competition level status (playing a sport in competition vs. playing a sport not in competition) did not interact with achievement values to predict physical activity in France and in China.

Our sixth hypothesis was that power is negatively associated with physical activity in China, but not in France. Our results tended to validate this hypothesis, as a greater attachment to power dominance was related to a lower level of physical activity in China, but not in France. This result is interesting as power dominance is motivationally opposed to benevolence values in the Schwartz model, and the results indicate that benevolence and benevolence care values in China positively predict physical activity. These results are congruent with our expectations and are related to prior research showing that the self in China is more interdependent and harmonious (Markus and Kitayama, 1991; Fiske and Taylor, 2008). The findings suggest that Chinese sport students are less likely to express power-dominance by engaging in physical activity. It might be the case that people who are perceived as dominant are more reluctant to engage in physical activities because it could have a negative impact on how they are perceived by others. Globally, this result confirms that Chinese individuals could associate strongly in memory physical activity with benevolence values (Hanel et al., 2018).

### Limitations and future directions

Several limitations of this work need to be mentioned. First, we did not measure objective but self-reported measures of physical activity, and participants tend to overestimate physical activity in self-reported measures (Nelson et al., 2019). However, as long as participants overestimated it to a similar extent, it does not threaten the validity of our findings. Previous research indicates that our measures are not impacted by socially desirable responding (Danioni and Barni, 2017), suggesting that our findings are probably not biased. Second, our participants were not representative of the whole populations both in France and in China, and we focused on sports science students. Consequently, the generalizability of the findings is limited. Future research with representative samples from France and China could add to the literature by also investigating whether our effects also generalize across a wider range of demographic groups (e.g., older participants and participants from different social backgrounds). Especially, following Skimina et al. (2019), it may be interesting in another study to understand the specific values (i.e., value states) participants have in mind when engaging in physical activity in the context of a sporting club. This may help to better understand the influence of achievement values (i.e., value traits) when engaging in physical activity in a competitive context.

Moreover, we did not focus on the gender impact on values–physical activity in this research. Future research can focus on how values may differently relate to physical activity according to gender. Crucially, it is important to explore what behaviors individuals in France and in China spontaneously associate with stimulation and benevolence values (e.g., Hanel et al., 2017). Finally, numerous studies have made use of the self-determination theory (Ryan and Deci, 2020) to understand physical activity (e.g., Teixeira et al., 2012). Recently, Vecchione and Schwartz (2022) have shown that values relate to autonomous and controlled forms of academic motivation to predict academic success. An interesting question and perspective for future research is to better understand how abstract values may relate to more specific forms of motivation (e.g., more or less autonomous or controlled) to predict physical activity.

## Conclusion

We found that conservation values were associated negatively with physical activity in similar ways in France and in China. In contrast, openness to change values were positively related to physical activity in France, but not in China. Similarly, benevolence values were related positively to physical activity in China, but not in France. Finally, the role of universalism and achievement values both in France and in China remained unclear in this research. Future research with representative samples from France and in China could clarify those relations.

## Data availability statement

The datasets presented in this study can be found in online repositories. The names of the repository/repositories and accession number(s) can be found in the article/Supplementary material.

## Ethics statement

The studies involving humans were approved by the University Committee on Human Research Protection of East China Normal University (HR 725–2021). The studies were conducted in accordance with the local legislation and institutional requirements. The participants provided their written informed consent to participate in this study. Written informed consent was obtained from the individual(s) for the publication of any potentially identifiable images or data included in this article.

## Author contributions

YL: Conceptualization, Data curation, Investigation, Software, Validation, Writing – original draft. OR: Investigation, Project administration, Supervision, Validation, Writing – review & editing. PH: Methodology, Supervision, Visualization, Writing – review & editing. JY: Supervision, Writing – review & editing. NS: Supervision, Writing – review & editing, Conceptualization, Data curation, Formal analysis, Project administration, Software, Writing – original draft.

## Footnotes

^1^https://scholarworks.gvsu.edu/orpc/vol2/iss2/9/
