# Supplementary material for: Values and physical activity among sports science students in France and China: a transcultural analysis
Source: Front Psychol. 2024 Jan 4;14:1304019. doi: 10.3389/fpsyg.2023.1304019 (PMC10794636; doi:10.3389/fpsyg.2023.1304019)
Supplement: Supplementary file 1 [file Data_Sheet_1.docx]

Table S1

*Conceptual definition of values*

| VALUES | CONCEPTUAL DEFINITION |
| --- | --- |
| Self-Direction |  |
| Self-direction-thought | Freedom to cultivate one’s own ideas and abilities |
| Self-direction-action | Freedom to determine one’s own actions |
| Stimulation | Excitement, novelty, and change |
| Hedonism | Pleasure and sensuous gratification |
| Achievement | Success according to social standards |
| Power |  |
| Power dominance | Power through exercising control over people |
| Power resources | Power through control of material and social resources |
| Face | Security and power through maintaining one’s public image and avoiding humiliation |
| Security |  |
| Security personal | Safety in one’s immediate environment |
| Security societal | Safety and stability in the wider society |
| Tradition | Maintaining and preserving cultural, family, or religious traditions |
| Conformity |  |
| Conformity rules | Compliance with rules, laws, and formal obligations |
| Conformity interpersonal | Avoidance of upsetting or harming other people |
| Humility | Recognizing one’s insignificance in the larger scheme of things |
| Benevolence |  |
| Benevolence dependability | Being a reliable and trustworthy member of the ingroup |
| Benevolence caring | Devotion to the welfare of ingroup members |
| Universalism |  |
| Universalism concern | Commitment to equality, justice, and protection for all people |
| Universalism nature | Preservation of the natural environment |
| Universalism tolerance | Acceptance and understanding of those who are different from oneself |

**Supplementary method**

**Participants.**

Three hundred and twenty eight participants stated that they did not make sporting competition (*M*_age_=20.24, *SD*=1.56; *M*_BMI_ =22.02, *SD*=2.43; 108 in France, *M*_age_=19.50, *SD*=2.02; *M*_BMI_ =21.96, *SD*=2.63; 220 in China, *M*_age_=20.60, *SD*=1.11; *M*_BMI_ =22.05, *SD*=2.32) and two hundred and ninety nine participants explained they made sporting competition (*M*_age_=19.18, *SD*=1.38; *M*_BMI_ =21.88, *SD*=2.33; 198 in France, *M*_age_=18.71, *SD*=1.31; *M*_BMI_ =21.96, *SD*=2.26, 101 in China, *M*_age_=20.11, *SD*=.99; *M*_BMI_ =21.73, *SD*=2.46).

Five participants in France stated that they made sporting competition at an international level (*M*_age_=18.40, *SD*=.54; *M*_BMI_ =20.35, *SD*=1.46, 0 in China) and six participants in France at a high national competition level (*M*_age_=18.50, *SD*=1.76; *M*_BMI_ =22.61, *SD*=2.38, 0 in China). In France and in China in combination, forty four participants stated they made sporting competition at a low national competition level (*M*_age_=19.00, *SD*=1.57; *M*_BMI_ =22.33, *SD*=2.71; 35 in France, *M*_age_=19.02, *SD*=1.52; *M*_BMI_ =22.36, *SD*=2.87, and 9 in China, *M*_age_=22.00, *SD*=1.41; *M*_BMI_ =24.59, *SD*=.14), one hundred and six at high regional competition level (*M*_age_=19.51, *SD*=1.24; *M*_BMI_ =21.89, *SD*=2.40; 47 in France, *M*_age_=18.51, *SD*=1.03; *M*_BMI_ =21.84, *SD*=1.86, and 59 in China (*M*_age_=20.12, *SD*=.93; *M*_BMI_ =21.88, *SD*=2.73), seventy-three at a low regional competition level (*M*_age_=19.32, *SD*=1.44; *M*_BMI_ =21.66, *SD*=2.11; 44 in France, *M*_age_=18.84, *SD*=1.42; *M*_BMI_ =22.00, *SD*=2.14, and 29 in China, *M*_age_=20.06, *SD*=1.04; *M*_BMI_ =21.27, *SD*=1.89), forty nine at a high departmental competition level (*M*_age_=18.73, *SD*=1.23; *M*_BMI_ =21.75, *SD*=2.31; 45 in France, *M*_age_=18.60, *SD*=1.26; *M*_BMI_ =21.73, *SD*=2.41, and 4 in China, *M*_age_=19.60, *SD*=.54; *M*_BMI_ =21.26, *SD*=1.53). Finally, sixteen participants in France stated they done competition level at a low departmental level (*M*_age_=18.75, *SD*=1.43; *M*_BMI_ =22.22, *SD*=1.74, 0 in China).

**Reliability.**

**CFA in higher order values.** We used confirmatory factor analysis (CFA) in France and in China to assess whether the 19 value types emerged. Due to the circular structure, we expected cross-loadings between opposing values in the circle. Such cross-loading are irrelevant for assessing whether neighboring values can be differentiated from one another. We therefore followed the common procedure for handling this in value research: i.e., we tested a separate model for each higher order value. Specifically, we used the Comparative Fit Index (CFI), the Root Mean Square Error of Approximation (RMSEA) to evaluate how our data fit the circular structure. We treated CFI≧.90, RMSEA ≦.08, and χ2/*df* <5 as indicating a reasonable model fit (Hu & Bentler, 1999).

**Measurement invariance.** We used multigroup CFA (MGCFA) to assess for measurement invariance. We expected both configural and metric invariance in France and in China. We did not expect to establish scalar invariance as scalar invariance is quite rarely established, especially in a complex value structure model (Cieciuch et al., 2016). We applied cutoffs (△CFI≦0.01 and △RMSEA≦0.015) proposed by Chen (2007) to determine measurement invariance between our French sample and our Chinese sample.

**Fit of the 19 values to the circular structure**. We used confirmatory multidimensional scaling (MDS; Borg et al., 2013) to assess the fit of the 19 values to the theorized circular structure in France and in China. MDS is commonly used for comparing the observed value circle with the theorized circle (e.g., Bilsky et al., 2011; Schwartz, 1992; Schwartz et al., 2012). We computed the value structure in France and in China, using the Torgerson initial configuration. We computed an MDS projection based on equally weighting the data from all groups to serve as the target structure.

We used Stress-I value to measure goodness-of-fit for the dimension, depended on two-dimension value structure and 19 items, stress-I belows to 0.269 implies an acceptable goodness-of-fit in diagram (Sturrock & Rocha, 2000). We report Tucker’s phi coefficient as

the index of congruence or similarity. Conventional rules of thumb for judging similarity consider the value of coefficients in the range of 0.85 to 0.94 corresponds to a fair similarity, a value higher than 0.95 implies two components can be considered equal (Lorenzo-Seva & Berge, 2006).

**Supplementary results**

**Reliability**

Table S2 presents the descriptive results in Chinese and French samples. In the French sample, all of the Cronbach’s alpha exceeded 0.60 in the four higher order, 14 of the Cronbach’s alpha exceeded 0.60 in the narrowly 19 values, while self-direction action, hedonism, achievement, face and humility were below 0.60. In the Chinese sample, all of the Cronbach’s alpha exceeded 0.60 in the four higher order, 16 of the coefficients exceeded 0.60 in the narrowly 19 values, while power dominance, security-personal and humility were below 0.60.

Table S2

*Descriptive results concerning values in France and in China*

|  | France | | | | | China | | | | |
| --- | --- | --- | --- | --- | --- | --- | --- | --- | --- | --- |
| MEASURES |  |  |  | Mean | |  |  |  | Mean | |
|  | N | Items | α | Raw | Centered | N | Items | α | Raw | Centered |
| SELF-TRANSCENDENCE | **308** | **15** | **.83** | **4.92** | **0.53** | **319** | **15** | **.88** | **4.96** | **0.19** |
| Universalism | 308 | 9 | .81 | 4.62 | 0.22 | 319 | 9 | .84 | 4.82 | 0.08 |
| Universalism-nature | 308 | 3 | .84 | 4.36 | -0.03 | 319 | 3 | .85 | 4.72 | -0.02 |
| Universalism-concern | 308 | 3 | .66 | 4.92 | 0.52 | 319 | 3 | .65 | 4.84 | 0.10 |
| Universalism-tolerance | 308 | 3 | .70 | 4.56 | 0.17 | 319 | 3 | .63 | 4.89 | 0.16 |
| Benevolence | 308 | 6 | .80 | 5.23 | 0.84 | 319 | 6 | .77 | 5.11 | 0.37 |
| Benevolence-care | 308 | 3 | .76 | 5.14 | 0.74 | 319 | 3 | .63 | 5.18 | 0.45 |
| Benevolence-dep | 308 | 3 | .71 | 5.33 | 0.93 | 319 | 3 | .61 | 5.03 | 0.29 |
| SELF-ENHANCEMENT | **308** | **9** | **.80** | **3.58** | **-0.81** | **319** | **9** | **.77** | **4.45** | **-0.44** |
| Power | 308 | 6 | .76 | 2.86 | -1.53 | 319 | 6 | .71 | 3.98 | -0.75 |
| Power resource | 308 | 3 | .74 | 3.14 | -1.25 | 319 | 3 | .62 | 3.80 | -0.94 |
| Power dominance | 308 | 3 | .69 | 2.58 | -1.81 | 319 | 3 | .58 | 4.17 | -0.57 |
| Achievement | 308 | 3 | .50 | 4.30 | -0.09 | 319 | 3 | .69 | 4.92 | 0.18 |
| OPENNESS TO CHANGE | **308** | **12** | **.75** | **4.94** | **0.54** | **319** | **12** | **.86** | **4.88** | **0.18** |
| Hedonism | 308 | 3 | .55 | 5.31 | 0.90 | 319 | 3 | .64 | 5.07 | 0.34 |
| Stimulation | 308 | 3 | .62 | 4.60 | 0.20 | 319 | 3 | .65 | 4.54 | -0.20 |
| Self-direction | 308 | 6 | .69 | 4.92 | 0.52 | 319 | 6 | .83 | 5.02 | 0.28 |
| Self-direction action | 308 | 3 | .47 | 4.85 | 0.59 | 319 | 3 | .69 | 4.88 | 0.14 |
| Self-direction though | 308 | 3 | .62 | 4.99 | 0.45 | 319 | 3 | .77 | 5.17 | 0.43 |
| CONSERVATION | **308** | **15** | **.83** | **3.97** | **-0.43** | **319** | **15** | **.83** | **4.60** | **-0.02** |
| Tradition | 308 | 3 | .75 | 3.45 | -0.94 | 319 | 3 | .73 | 4.04 | -0.70 |
| Security | 308 | 6 | .79 | 4.64 | 0.24 | 319 | 6 | .76 | 5.24 | 0.50 |
| Security person | 308 | 3 | .60 | 4.64 | 0.24 | 319 | 3 | .59 | 5.19 | 0.45 |
| Security societal | 308 | 3 | .78 | 4.63 | 0.24 | 319 | 3 | .75 | 5.29 | 0.55 |
| Conformity | 308 | 6 | .79 | 3.81 | -0.58 | 319 | 6 | .74 | 4.52 | -0.22 |
| Conformity inter | 308 | 3 | .82 | 3.84 | -0.56 | 319 | 3 | .72 | 4.78 | 0.04 |
| Conformity rules | 308 | 3 | .81 | 3.79 | -0.60 | 319 | 3 | .74 | 4.26 | -0.48 |
| Humility | 308 | 3 | .28 | 4.32 | -0.07 | 319 | 3 | .41 | 4.44 | -0.30 |
| Face | 308 | 3 | .56 | 4.46 | 0.07 | 319 | 3 | .72 | 4.75 | 0.01 |
| PHYSICAL ACTIVITY |  |  |  |  |  |  |  |  |  |  |
| Work | 308 |  |  | 3874.2 |  | 319 |  |  | 3940 |  |
| Transport | 308 |  |  | 1088.6 |  | 319 |  |  | 520.7 |  |
| Housework | 308 |  |  | 770.8 |  | 319 |  |  | 628.9 |  |
| Leisure | 308 |  |  | 3698.5 |  | 319 |  |  | 2223.2 |  |
| GLOBAL PA | 308 |  |  | 9432.2 |  | 319 |  |  | 7312.9 |  |

*Note*. Work=physical activity in job-related domain, Transport=physical activity in transportation domain, House=physical activity in domestic domain, Leisure=physical activity in leisure-time domain, Global=total physical activity in four different domains.

**Measurement models within groups: CFA**

We followed the Schwartz’s rule, assigning three values humility, face, and hedonism which are located on the border between two higher order values in the value circle. We assigned the value to the higher order value with which it correlated more highly in Schwartz’s study (Schwartz, 2021). We correlated each of the three values with its two neighbouring higher order values in each of the 2 cultural groups. This led to assigning face to conservation, hedonism to openness to change, and humility to self-transcendence. To evaluated the differentiation of the 19 values within each cultural group, we examined the fit coefficients of the CFA for each higher order set of adjacent value (see Table S2 for a summary).

As shown in Table S3, the measurement models for self-transcendence, openness to change, and conservation were acceptable according to model fit index. Only self-enhancement was problematic. To understand the problem, we then examined the modification indexes. The model modification indexes indicated there were three errors correlations between “por3” to “pod1”, “por3” to “pod2”, as well as” por3” and “pod3”. After the model modification, self-enhancement also showed an acceptable model fit in the China sample (*χ2/df*=3.383, CFI=0.920, RMSEA=0.08).

Table S3

*Model fit for the given measurement in Portrait Value Questionnaire*

|  | China | | | France | | |
| --- | --- | --- | --- | --- | --- | --- |
| MEASURES | χ2/*df* | χ2/*df* | χ2/*df* | χ2/*df* | CFI | RMSEA |
| Self-Transcendence (unn,unc,unt,bec,bed) | 2.27 | 1.30 | 1.30 | 1.30 | 0.93 | .063 |
| Self-Enhancement (ach, por, pod) | 4.92 | 2.82 | 2.82 | 2.82 | 0.84 | .111 |
| Openness to change (sdt, sta, sti, hed) | 2.83 | 1.59 | 1.59 | 1.59 | 0.93 | .076 |
| Conservation (tra, cor, coi, sep,ses) | 2.19 | 2.27 | 2.27 | 2.27 | 0.92 | .061 |

**Measurement invariance across group: MGCFA**

We ran an MGCFA for each of the four higher order and applied the cut-offs of Chen (2007) to test for the measurement invariance of values across France and China. Table S4 summarizes the findings for each level of invariance (configural, metric, and scalar) for each higher order value. Measurement invariance were established across the two sample for self-transcendence, openness to change, and conservation. The original self-enhancement model failed to support both configural and metric invariance because the model did not meet the threshold for RMSEA (Original model: △CFI metric model=0.021, △CFI scalar model=0.408). Introducing three model modifications (“pod3” to “por1”, “pod3” to “por2”, “pod3” to “por1”) improved the model fitness for self-enhancement, making it acceptable at the configural level and nearly acceptable at the metric level.

Table S4

*Measurement invariance of each higher order value*

| **MEASURES** | χ2 (*df*) | CFI | RMSEA | △χ2 (*df*) | △CFI | △RMSEA |
| --- | --- | --- | --- | --- | --- | --- |
| **Self-transcendece** | | | | | | |
| configural | 355.21 (160)^**^ | 0.93 | 0.04 |  |  |  |
| metric | 387.55 (166)^**^ | 0.92 | 0.04 | 32.33 (6)^**^ | 0.01 | .002 |
| scalar | 579.26 (175^**^ | 0.86 | 0.06 | 224.04 (15)^**^ | 0.09 | .019 |
| **self-enhancement (modified)** | | | | | |  |
| configural | 129.90 (42)^**^ | 0.93 | 0.06 |  |  |  |
| metric | 161.15 (48)^**^ | 0.91 | 0.07 | 19.12 (6)^**^ | 0.02 | .003 |
| scalar | 183.97 (54^**^ | 0.89 | 0.12 | 30.46 (12)^**^ | 0.02 | .004 |
| **openness to change** | | | | | | |
| configural | 212.76 (96)^**^ | 0.93 | 0.04 |  |  |  |
| metric | 226.82 (104)^**^ | 0.93 | 0.04 | 14.05 (8) | 0.01 | .001 |
| scalar | 583.98 (116)^**^ | 0.75 | 0.08 | 371.21 (20)^**^ | 0.18 | .037 |
| **Conservation** | | | | | | |
| configural | 357.53 (160)^**^ | 0.93 | 0.04 |  |  |  |
| metric | 369.57 (168)^**^ | 0.93 | 0.04 | 12.04 (8) | 0.01 | <.001 |
| scalar | 680.73 (180)^**^ | 0.83 | 0.06 | 323.2 (20)^**^ | 0.10 | .023 |

*Note*. **p*<.05 ; ***p*<.01

**Circular structure of values: MDS**

Figure 1S and Figure 2S present the MDS two-dimensional projection of 19 centered values based on the pooled correlation matrix in France and in China. The order of values around circle in this overall structure corresponds to the theorized order with no reversals. In the structure of value in the French sample, the indication of Stress-I (0.167) and Tucker’s phi coefficient (0.986) were acceptable to the thresholds. Openness to change values can be seen in the top left of Figure S1, self-enhancement values in the top right, self-transcendence values in the lower left, the conservation values on the lower right. Contrary to the theoretical structure (Schwartz et al., 2012), benevolence-care and benevolence-dependability were closer to openness to change.

For the Chinese sample, Stress-I (0.206) and Tucker’s phi coefficient (0.979) were acceptable to the thresholds. Also, openness to change values were on the lower left, self-enhancement values on the top left, the self-transcendence values on the lower right, and conservation values on the top right. However, security was far away from other conservation values, as well as benevolence was far away from other self-transcendence values.


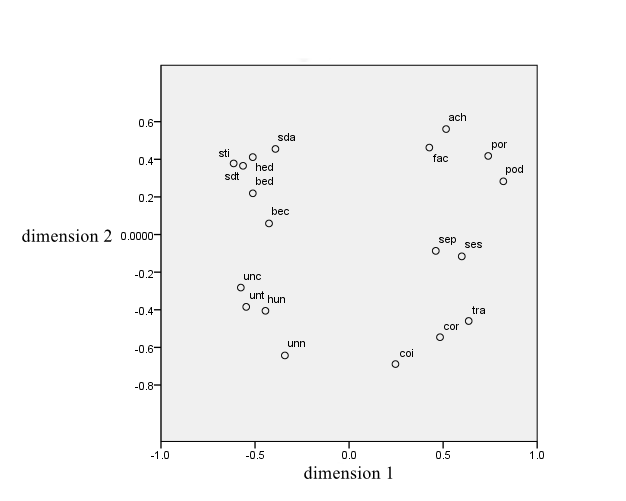


Figure S1. Values structure in French participants


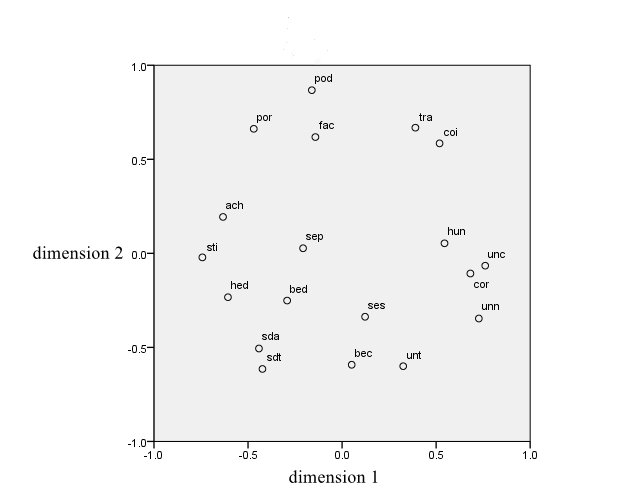


Figure S2. Values structure in Chinese participants

**Supplementary descriptive statistics**

**Four higher domains of values**. The importance of the four higher category of values was analyzed with a 2 x 4 (Type of country X higher order values) mixed ANOVA, with Country (France vs. China) between-subjects factor and Higher order values (self-transcendence vs. self-enhancement vs. openness to change vs. conservation) as within-subjects factor. Results indicated a significant main effect of country, *F*(1,625)=5.07, *p*=.024, of higher order value type, *F*(3,1875)=529.91, *p*<.001, and a significant two-way interaction between country and higher order, *F*(3,1875)=28.53, *p*<.001.

The significant two-way interaction (see TableS5) between country and higher order indicates that participants attached more importance to self-transcendence values and openness to change values in France than in China (*p*<.001). Also, they attached both in France and in China more importance to self-transcendence and openness to change values in references to conservatism and self-enhancement values, *ps*<.001. In France and in China, participants attached more importance to conservation values than to self-enhancement values, *ps*<.001.

Interestingly, French participants attached more importance to self-transcendence values than Chinese participants, *p*<.001. Also, French participants attached more importance to openness to change values than Chinese participants, *p*<.001. Furthermore, Chinese participants attached more importance to conservatism values than French participants, *p*<.001. Also, Chinese participants attached more importance to self-enhancement values than French participants, *p*<.001.

**Ten categories of values**. The attachment to the ten value types was analyzed with a 2 x 10 (Type of country X broader values) mixed ANOVA. Results indicated a non-significant effect of country, *F*(1,625)=1.77, *p*=.182, a significant main effect of broader categories, *F*(9,5625)=448.25, *p*<.001, and a significant two-way interaction between country and broader categories, *F*(9,5625)=60.23, *p*<.001.

The significant two-way interaction (see Table S6 and S7) between country and value types indicates that self-direction, stimulation, hedonism, universalism and benevolence values were more important in France than in China, *ps* <.001. Achievement, power, security, tradition and conformity were more important in China than in France, *ps*<.001. The order of value importance was also different across countries.

In France, hedonism and benevolence values were the most important values. Participants attached a similar level of importance to hedonism as to benevolence (*p*=.195), which were both in turn more important than self-direction values, *ps* <.001. Self-Direction values were more important than security, universalism, and stimulation values, *ps* <.001. But security values were as important as universalism values, *p*=.718, and universalism values were as important as stimulation values, *p*=.793. Stimulation values were more important than achievement values, *p*<.001, which in turn were more important than conformity values, *p*<.001. Conformity values were more important than tradition values, p<.001, which in turn were more important than power values, *p*<.001.

In China, security values were most important. Security values were more important than benevolence values, *p*=.017. But benevolence values were as important as hedonism values, *p*=.585, which were in turn as important as self-direction values, *p*=.354. Self-direction values were marginally more important than achievement values, *p*=.067.

Achievement values were marginally more important than universalism values, *p*=.062, which were in turn more important than stimulation values, *p*<.001. Stimulation values were as important as conformity values, *p*=.718, which were in turn more important than tradition values, *p*<.001. Finally, tradition values were as important as power values, *p*=.291.

Table S5

*Results of moderated regressions for higher order values predicting global physical activity, moderated by country*

|  |  | Global Physical activity | | |
| --- | --- | --- | --- | --- |
|  |  | T | *p* | *B* |
| Openness to change | R²=.036, *F*(3,623)=8.99, *p*<.001 | | | |
|  | Openness to change | 2.65 | .006 | .11 |
|  | Country | 2.75 | .008 | .11 |
|  | Openness to change X country | 1.18 | .236 | .04 |
|  |  |  |  |  |
|  | R²=.106, *F*(5,621)=15.87, *p*<.001 | | | |
|  | Gender | 2.60 | .009 | .10 |
|  | Age | .01 | .992 | .00 |
|  | Country | 1.02 | .305 | .04 |
|  | Competition level | 6.64 | <.001 | .26 |
|  | Openness to change | 3.02 | .002 | .12 |
|  |  |  |  |  |
| Self-Enhancement | R²=.025, *F*(3,623)=6.49, *p*<.001 | | | |
|  | Self-Enhancement | .14 | .882 | .01 |
|  | Country | 3.97 | <.001 | .16 |
|  | Self-Enhancement X country | 1.51 | .131 | .06 |
| Conservatism | R²=.022, *F*(3,623)=5.76, *p*<.001 | | | |
|  | Conservatism | -.38 | .696 | -.02 |
|  | Country | 3.43 | <.001 | .15 |
|  | Conservatism X country | -.31 | .753 | -.01 |
|  |  |  |  |  |
| Self-Transcendence | R²=.022, *F*(3,623)=5.80, *p*<.001 | | | |
|  | Self-Transcendence | -.45 | .652 | -.02 |
|  | Country | 3.89 | <.001 | .17 |
|  | Self-Transcendence X country | -.51 | .608 | -.02 |

*Note*. Gender: -.05 for women and +.05 for men, Country: -.05 for France and +.05 for China.

Table S6

*Results of moderated regressions for Self-Direction, Stimulation, Hedonism, Achievement, Power values with country as moderator in predicting global physical activity*

|  |  | Global Physical activity | | |
| --- | --- | --- | --- | --- |
|  |  | T | *p* | *B* |
| Self-Direction | R²=.024, *F*(3,623)=6.14, *p*<.001 | | | |
|  | Self-Direction | .34 | .730 | .01 |
|  | Country | 3.91 | <.001 | .16 |
|  | Self-Direction X Country | -1.25 | .211 | -.05 |
| *Self-Direction-Though* R²=.022, *F*(3,623)=5.84, *p*<.001 | | | | |
|  | Self-Direction-Though | .83 | .401 | .03 |
|  | Country | 3.77 | <.001 | .15 |
|  | Self-Direction-Though X Country | .03 | .973 | .00 |
| Universalism | R²=.026, *F*(3,623)=6.23, *p*<.001 | | | |
|  | Universalism | -1.15 | .265 | -.06 |
|  | Country | 4.21 | <.001 | .17 |
|  | Universalism X Country | 1.57 | .116 | .06 |
| *Universalism-Tolerance* R²=.021, *F*(3,623)=5.61, *p*<.001 | | | | |
|  | Universalism-Tolerance | -.08 | .935 | .00 |
|  | Country | 4.10 | <.001 | .16 |
|  | Universalism-Tolerance X Country | -.10 | .919 | .00 |
| *Universalism-Concern* R²=.028, *F*(3,623)=7.07, *p*<.001 | | | | |
|  | Universalism-Concern | -2.07 | .038 | -.09 |
|  | Country | 4.55 | <.001 | .19 |
|  | Universalism-Concern X Country | .28 | .796 | .01 |
|  | R²=.094, *F*(5,621)=14.07, *p*<.001 | | | |
|  | Gender | 2.42 | .015 | .09 |
|  | Age | .05 | .952 | .00 |
|  | Country | 2.40 | .016 | .10 |
|  | Competition level | 6.45 | <.001 | .26 |
|  | Universalism-Concern | -1.02 | .305 | -.04 |
| *Benevolence-Dependability* R²=.032, *F*(3,623)=7.92, *p*<.001 | | | | |
|  | Benevolence-Dependability | .19 | .843 | .01 |
|  | Country | 3.43 | <.001 | .16 |
|  | Ben-Dependability X Country | -2.59 | .009 | -.10 |
| France | R²=.062, *F*(4,303)=6.11, *p*<.001 | | | |
|  | Gender | 1.94 | .053 | .11 |
|  | Age | 1.26 | .207 | .07 |
|  | Competition level | 4.08 | <.001 | .23 |
|  | Ben-Dependability | -1.55 | .121 | -.09 |
| China | R²=.103, *F*(4,314)=10.17, *p*<.001 | | | |
|  | Gender | 2.06 | .040 | .11 |
|  | Age | -1.94 | .052 | -.11 |
|  | Competition level | 5.17 | <.001 | .28 |
|  | Ben-Dependability | 1.31 | .188 | .07 |

*Note*. Gender: -.05 for women and +.05 for men, Country: -.05 for France and +.05 for China.

Table S7

*Results of moderated regressions for Conformity, Security, Tradition, Humility, Face, Power, Achievement values with country as moderator in predicting global physical activity*

|  |  | Global Physical activity | | |
| --- | --- | --- | --- | --- |
|  |  | T | *p* | *B* |
| Conformity | R²=.028, *F*(3,623)=7.21, *p*<.001 | | | |
|  | Conformity | -1.36 | .173 | -.05 |
|  | Country | 3.61 | <.001 | .15 |
|  | Conformity X Country | -1.20 | .229 | -.05 |
| Conformity-Interpersonal R²=.023, *F*(3,623)=6.08, *p*<.001 | | | | |
|  | Conformity-Inter | -.47 | .633 | -.02 |
|  | Country | 4.08 | <.001 | .16 |
|  | Conformity-Inter X Country | -.98 | .327 | -.04 |
| *Security societal* | R²=.024, *F*(3,623)=6.27, *p*<.001 | | | |
|  | Security societal | -1.37 | .169 | -.06 |
|  | Country | 3.71 | <.001 | .15 |
|  | Security societal X country | .16 | .868 | .01 |
| Tradition | R²=.029, *F*(3,623)=7.29, *p*<.001 | | | |
|  | Tradition | 1.63 | .103 | .06 |
|  | Country | 4.29 | <.001 | .17 |
|  | Tradition X Country | 1.18 | .234 | .05 |
| Humility | R²=.022, *F*(3,623)=5.89, *p*<.001 | | | |
|  | Humility | -.83 | .401 | -.03 |
|  | Country | 4.18 | <.001 | .16 |
|  | Humility X Country | .42 | .670 | .02 |
| Face | R²=.027, *F*(3,623)=6.89, *p*<.001 | | | |
|  | Face | -1.54 | .122 | -.06 |
|  | Country | 4.17 | <.001 | .16 |
|  | Face X Country | -1.03 | .301 | -.04 |
| Power | R²=.025, *F*(3,623)=6.38, *p*<.001 | | | |
|  | Power | -.84 | .398 | -.04 |
|  | Country | 3.30 | <.001 | .14 |
|  | Power X Country | 1.36 | .172 | .05 |
| *Power-Resources* | R²=.022, *F*(3,623)=5.80, *p*<.001 | | | |
|  | Power Resources | .07 | .940 | .00 |
|  | Country | 3.89 | <.001 | .16 |
|  | Power Resources X Country | -.77 | .441 | -.03 |
| Achievement | R²=.031, *F*(3,623)=7.77, *p*<.001 | | | |
|  | Achievement | 2.36 | .018 | .10 |
|  | Country | 4.54 | <.001 | .18 |
|  | Achievement X country | -.002 | .998 | .00 |
|  | R²=.096, *F*(5,621)=14.41, *p*<.001 | | | |
|  | Gender | 2.31 | .020 | .09 |
|  | Age | -.00 | .995 | -.01 |
|  | Country | 2.46 | .014 | .11 |
|  | Competition level | 6.42 | <.001 | .26 |
|  | Achievement | 1.59 | .110 | .06 |

*Note*. Gender: -.05 for women and +.05 for men, Country: -.05 for France and +.05 for China.

Table S8

*Interrelations between four higher order values in France*

|  | Transcendence | Enhancement | Openness to change | Conservation |
| --- | --- | --- | --- | --- |
| Transcendence | - |  |  |  |
| Enhancement | -.53^**^ | - |  |  |
| Openness to change | .20^**^ | -.15^**^ | - |  |
| Conservation | -.35^**^ | -.24^**^ | -.54^**^ | - |

*Note*. **p*<.05 ; ***p*<.01

Table S9

*Interrelations between the 10 value types in France*

|  | SD | Sti | Hed | Ach | Pow | Sec | Con | Tra | Ben | Uni |
| --- | --- | --- | --- | --- | --- | --- | --- | --- | --- | --- |
| Self-direction | - |  |  |  |  |  |  |  |  |  |
| Stimulation | .23^**^ | - |  |  |  |  |  |  |  |  |
| Hedonism | .22^**^ | .25^**^ | - |  |  |  |  |  |  |  |
| Achievement | -.09 | -.10 | -.05 | - |  |  |  |  |  |  |
| Power | -.07 | -.15^*^ | -.06 | .49^**^ | - |  |  |  |  |  |
| Security | -.22^**^ | -.39^**^ | -.20^**^ | -.04 | -.01 | - |  |  |  |  |
| Conformity | -.37^**^ | -.35^**^ | -.35^**^ | -.22^**^ | -.29^**^ | .13^*^ | - |  |  |  |
| Tradition | -.16^**^ | -.06 | -.17^**^ | -.09 | -.09 | .08 | .01 | - |  |  |
| Benevolence | .06 | .04 | .26^**^ | -.07 | -.30^**^ | -.05 | -.23^**^ | -.04 | - |  |
| Universalism | .04 | .17^**^ | 0.04 | -.40^**^ | -.46^**^ | -.30^**^ | -.01 | -.27^**^ | .02 | - |

*Note*. **p*<.05 ; ***p*<.01; SD=self-direction, Sti=stimulation, Hed=hedonism, Ach=achievement, Pow=power, Sec=security, Con=conformity, Tra=tradition, Ben=benevolence, Un=university.

Table S10

*Interrelations between the 19 value types in France*

|  | Sdt | Sda | Sti | Hed | Ach | Pod | Por | Fac | Sep | Ses | Tra | Cor | Coi | Hum | Unn | Unc | Unt | Bec | Bed |
| --- | --- | --- | --- | --- | --- | --- | --- | --- | --- | --- | --- | --- | --- | --- | --- | --- | --- | --- | --- |
| Sdt | - |  |  |  |  |  |  |  |  |  |  |  |  |  |  |  |  |  |  |
| Sda | .45^**^ | - |  |  |  |  |  |  |  |  |  |  |  |  |  |  |  |  |  |
| Sti | .21^**^ | .17^**^ | - |  |  |  |  |  |  |  |  |  |  |  |  |  |  |  |  |
| Hed | .13^*^ | .23^**^ | .25^**^ | - |  |  |  |  |  |  |  |  |  |  |  |  |  |  |  |
| Ach | -.10 | -.05 | -.10 | -.05 | - |  |  |  |  |  |  |  |  |  |  |  |  |  |  |
| Pod | .08 | -.06 | .00 | -.08 | .35^**^ | - |  |  |  |  |  |  |  |  |  |  |  |  |  |
| Por | -.17^**^ | -.05 | -.23^**^ | -.02 | .46^**^ | .36^**^ | - |  |  |  |  |  |  |  |  |  |  |  |  |
| Fac | -.15^**^ | -.04 | -.20^**^ | -.08 | .24^**^ | .06 | .24^**^ | - |  |  |  |  |  |  |  |  |  |  |  |
| Sep | -.18^**^ | -.04 | -.32^**^ | -.18^**^ | -.02 | -.17^**^ | -.01 | .08 | - |  |  |  |  |  |  |  |  |  |  |
| Ses | -.16^**^ | -.20^**^ | -.30^**^ | -.14^*^ | -.04 | .00 | 0.11 | -.03 | .29^**^ | - |  |  |  |  |  |  |  |  |  |
| Tra | -.13^*^ | -.14^*^ | -.06 | -.17^**^ | -.09 | -.05 | -.09 | -.12^*^ | .05 | .07 | - |  |  |  |  |  |  |  |  |
| Cor | -.23^**^ | -.26^**^ | -.29^**^ | -.38^**^ | -.21^**^ | -.20^**^ | -.14^*^ | -.07 | .24^**^ | .18^**^ | .10 | - |  |  |  |  |  |  |  |
| Coi | -.23^**^ | -.26^**^ | -.25^**^ | -.18^**^ | -.13^*^ | -.15^**^ | -.24^**^ | .00 | -.04 | -.05 | -.08 | .19^**^ | - |  |  |  |  |  |  |
| Hum | .02 | .06 | .08 | .00 | -.37^**^ | -.23^**^ | -.35^**^ | -.15^**^ | -.09 | -.15^**^ | -.02 | -.05 | .06 | - |  |  |  |  |  |
| Unn | .04 | -.07 | .19^**^ | -.01 | -.23^**^ | -.18^**^ | -.23^**^ | -.22^**^ | -.17^**^ | -.17^**^ | -.21^**^ | -.09 | -.02 | .08 | - |  |  |  |  |
| Unc | .02 | -.03 | .07 | .05 | -.35^**^ | -.29^**^ | -.27^**^ | -.17^**^ | -.17^**^ | -.11 | -.22^**^ | -.10 | .04 | .16^**^ | .27^**^ | - |  |  |  |
| Unt | .10 | .09 | .08 | .06 | -.32^**^ | -.31^**^ | -.37^**^ | -.28^**^ | -.16^**^ | -.26^**^ | -.15^**^ | .02 | .11^*^ | .14^*^ | .16^**^ | .38^**^ | - |  |  |
| Bec | .01 | .07 | .04 | .21^**^ | -.09 | -.27^**^ | -.19^**^ | .04 | -.01 | -.06 | .00 | -.16^**^ | -.19^**^ | .08 | -.07 | .15^**^ | .05 | - |  |
| Bed | .00 | .09 | .03 | .20^**^ | -.01 | -.19^**^ | -.14* | .00 | .02 | -.06 | -.08 | -.10 | -.10 | -.09 | -.13^*^ | .11 | .01 | .28^**^ | - |

*Note.* **p*<.05 ; ***p*<.01; Sdt=self-direction-thought, Sda=self-direction-action, Sti=stimulation, hed=hedonism, Ach=achievement, Pod=power-dominance, Por=power-resources, Fac=face, Sep=security-personal, Ses=security-societal, Tra=tradition, Cor=conformity-rules, Coi=conformity-interpersonal, Hum=humility, Unc=university-concern, Unn=universalism-nature, Unt=universalism-tolerance, Bec=benecolence-caring, Bed=benevolence-dependability.

Table S11

*Interrelations between four higher order values in China*

|  | Transcendence | Enhancement | Openness to change | Conservation |
| --- | --- | --- | --- | --- |
| Transcendence | - |  |  |  |
| Enhancement | -.59^**^ | - |  |  |
| Openness to change | -.20^**^ | -.06 | - |  |
| Conservation | -.13^*^ | -.27^**^ | -.60^**^ | - |

*Note*. **p*<.05 ; ***p*<.01

Table S12

*Interrelations between 10 value types in China*

|  | SD | Sti | Hed | Ach | Pow | Sec | Con | Tra | Ben | Uni |
| --- | --- | --- | --- | --- | --- | --- | --- | --- | --- | --- |
| Self-direction | - |  |  |  |  |  |  |  |  |  |
| Stimulation | .18^**^ | - |  |  |  |  |  |  |  |  |
| Hedonism | .24^**^ | .14* | - |  |  |  |  |  |  |  |
| Achievement | .18^**^ | .12* | .09 | - |  |  |  |  |  |  |
| Power | -.20^**^ | .04 | -.07 | .07 | - |  |  |  |  |  |
| Security | .04 | -.17** | .09 | -.03 | -.21** | - |  |  |  |  |
| Conformity | -.34^**^ | -.37** | -.26** | -.25** | -.16** | -.12* | - |  |  |  |
| Tradition | -.30^**^ | -.19** | -.25** | -.12* | .07 | -.28** | .05 | - |  |  |
| Benevolence | .04 | -.10 | .06 | -.07 | -.29** | .19** | -.21** | -.21** | - |  |
| Universalism | -.12^*^ | -.13* | -.24** | -.19** | -.47** | -.12* | .07 | -.04 | -.07 | - |

*Note*. **p*<.05 ; ***p*<.01; SD=self-direction, Sti=stimulation, Hed=hedonism, Ach=achievement, Pow=power, Sec=security, Con=conformity, Tra=tradition, Ben=benevolence, Uni=universalism

Table S13

|  | Sdt | Sda | Sti | Hed | Ach | Pod | Por | Fac | Sep | Ses | Tra | Cor | Coi | Hum | Unn | Unc | Unt | Bec | Bed |
| --- | --- | --- | --- | --- | --- | --- | --- | --- | --- | --- | --- | --- | --- | --- | --- | --- | --- | --- | --- |
| Sdt | - |  |  |  |  |  |  |  |  |  |  |  |  |  |  |  |  |  |  |
| Sda | .36^**^ | - |  |  |  |  |  |  |  |  |  |  |  |  |  |  |  |  |  |
| Sti | .15^**^ | .15^**^ | - |  |  |  |  |  |  |  |  |  |  |  |  |  |  |  |  |
| Hed | .15^**^ | .24^**^ | .14^*^ | - |  |  |  |  |  |  |  |  |  |  |  |  |  |  |  |
| Ach | .14^*^ | .16^**^ | .12^*^ | .09 | - |  |  |  |  |  |  |  |  |  |  |  |  |  |  |
| Pod | -.17^**^ | -.22^**^ | .08 | -.13* | -.06 | - |  |  |  |  |  |  |  |  |  |  |  |  |  |
| Por | -.10 | -.07 | -.01 | -.00 | .18** | .35** | - |  |  |  |  |  |  |  |  |  |  |  |  |
| Fac | -.25^**^ | -.21^**^ | -.08 | -.14* | .13* | .04 | .17** | - |  |  |  |  |  |  |  |  |  |  |  |
| Sep | -.10 | .17^**^ | -.16^**^ | .07 | .06 | -.18** | -.04 | .02 | - |  |  |  |  |  |  |  |  |  |  |
| Ses | .00 | .04 | -.10 | .06 | -.10 | -.16** | -.11 | .03 | .06 | - |  |  |  |  |  |  |  |  |  |
| Tra | -.18^**^ | -.32^**^ | -.19^**^ | -.25** | -.12* | .09 | .03 | -.02 | -.11* | -.28** | - |  |  |  |  |  |  |  |  |
| Cor | -.07 | -.06 | -.28^**^ | -.25** | -.15** | -.18** | -.30** | -0.11 | .06 | -.03 | .03 | - |  |  |  |  |  |  |  |
| Coi | -.30^**^ | -.35^**^ | -.27^**^ | -.15** | -.21** | .07 | -.02 | .14* | -.10 | -.15** | .05 | .07 | - |  |  |  |  |  |  |
| Hum | -.05 | -.16^**^ | -0.1 | -.08 | -.41** | -0.10 | -.27** | -.19** | -.11* | -.03 | .11 | .03 | .07 | - |  |  |  |  |  |
| Unn | -.08 | -.15^**^ | -.08 | -.24** | -.07 | -.22** | -.30** | -.18** | -.12* | .01 | .02 | .31** | -.14* | -.06 | - |  |  |  |  |
| Unc | -.22^**^ | -.19^**^ | -.16^**^ | -.14* | -.19** | -.19** | -.34** | -.07 | -.18** | .07 | -.05 | .12* | .05 | .13* | .31** | - |  |  |  |
| Unt | .19^**^ | .06 | -.01 | -.07 | -.13* | -.18** | -.32** | -.33** | -.14* | -.03 | -.07 | -.03 | -.06 | .12* | .06 | .09 | - |  |  |
| Bec | -.02 | .10 | -.15^**^ | .02 | -.10 | -.27** | -.17** | -.10 | .18** | .14* | -.18** | -.09 | -.15** | .04 | -.03 | .01 | .06 | - |  |
| Bed | -.05 | .08 | .00 | .07 | -.01 | -.22** | -.12* | .13* | .16** | -.02 | -.16** | -.17** | -.09 | -.04 | -.21** | -.03 | .01 | .26** | - |

*Interrelations between 19 value types in China*

*Note.* **p*<.05 ; ***p*<.01; Sdt=self-direction-thought, Sda=self-direction-action, Sti=stimulation, hed=hedonism, Ach=achievement, Pod=power-dominance, Por=power-resources, Fac=face, Sep=security-personal, Ses=security-societal, Tra=tradition, Cor=conformity-rules, Coi=conformity-interpersonal, Hum=humility, Unc=university-concern, Unn=universalism-nature, Unt=universalism-tolerance, Bec=benecolence-caring, Bed=benevolence-dependability.

Table S14

*Interrelations between values and physical activity in the different domains*

|  | France (N=308) | | | | | China (N=319) | | | | |
| --- | --- | --- | --- | --- | --- | --- | --- | --- | --- | --- |
| MEASURES | Work | Transport | House | Leisure | Global | Work | Transport | House | Leisure | Global |
| **SELF-T** | .07 | .06 | -.02 | -.14^*^ | -.05 | .01 | .08 | -.01 | -.02 | .00 |
| Universalism | .06 | .16^**^ | .04 | -.09 | .02 | -.07 | .07 | -.05 | -.11^*^ | -.09 |
| Unn | .06 | .16^**^ | .09 | .03 | .10 | -.07 | .04 | .01 | -.13^*^ | -.09 |
| Unc | .03 | .05 | -.04 | -.16^**^ | -.08 | -.05 | .01 | -.15^**^ | -.07 | -.09 |
| Unt | .04 | .12^*^ | .01 | -.10 | -.01 | -.01 | .09 | .02 | -.01 | .00 |
| Benevolence | .03 | -.11^*^ | -.08 | -.12^*^ | -.10 | .12^*^ | .05 | .07 | .14^**^ | .15^**^ |
| Bec | .05 | -.14^*^ | -.06 | -.08 | -.07 | .12^*^ | .05 | .11 | .09 | .14^*^ |
| Bed | -.01 | -.04 | -.08 | -.12^*^ | -.10 | .07 | .02 | .01 | .14^*^ | .11 |
| **SELF-E** | -.02 | -.04 | .02 | .1^5**^ | .08 | -.05 | -.09 | .02 | -.03 | -.05 |
| Power | -.02 | -.07 | -.01 | .08 | .02 | -.08 | -.09 | .00 | -.05 | -.08 |
| Por | -.06 | -.09 | -.08 | .05 | -.03 | .02 | -.10 | .04 | .06 | .03 |
| Pod | .02 | -.02 | .07 | .09 | .07 | -.15^**^ | -.04 | -.04 | -.15^**^ | -.17^**^ |
| Achievement | -.004 | .02 | .04 | .18^**^ | .12^*^ | .08 | -.04 | .05 | .05 | .08 |
| **OPEN** | .14^*^ | .16^**^ | .06 | .09 | .17^**^ | .04 | .03 | .09 | .02 | .05 |
| Hedonism | .11^*^ | .12^*^ | .00 | .06 | .12^*^ | .08 | .01 | -.03 | -.02 | .04 |
| Stimulation | .16^**^ | .16^**^ | .15^**^ | .15^**^ | .24^**^ | -.00 | .02 | .11 | -.04 | .01 |
| Self-direction | -.01 | .04 | -.06 | -.05 | -.04 | .01 | .02 | .09 | .08 | .06 |
| Sda | -.07 | -.02 | -.09 | -.08 | -.10 | .04 | .02 | .04 | .08 | .07 |
| Sdt | .05 | .09 | -.02 | -.01 | .04 | -.02 | .02 | .11 | .05 | .03 |
| **CONS** | -.08 | -.08 | -.02 | .04 | -.04 | -.01 | -.04 | -.08 | .05 | -.00 |
| Tradition | -.03 | .02 | .11^*^ | .19^**^ | .13^*^ | -.04 | .05 | .05 | .06 | .02 |
| Security | -.08 | -.18^**^ | -.18^**^ | -.08 | -.16^**^ | .01 | -.10 | -.11^*^ | .02 | -.01 |
| Sep | -.14^*^ | -.22^**^ | -.18^**^ | -.10 | -.22^**^ | .07 | .00 | -.07 | .02 | .04 |
| Ses | -.00 | -.08 | -.11 | -.04 | -.06 | -.05 | -.13^*^ | -.09 | .01 | -.05 |
| Conformity | -.06 | -.05 | -.05 | -.12^*^ | -.13^*^ | .01 | -.03 | -.07 | .00 | -.01 |
| Cor | -.05 | -.13^*^ | -.09 | -.10 | -.13^*^ | .01 | -.02 | -.06 | -.05 | -.03 |
| Coi | -.05 | .05 | .01 | -.09 | -.07 | .02 | -.02 | -.04 | .04 | .02 |
| Humility | -.04 | .08 | .06 | -.04 | -.02 | -.05 | .06 | -.02 | -.04 | -.05 |
| Face | -.03 | -.12^*^ | -.10 | -.09 | -.11 | .04 | -.08 | -.09 | -.04 | -.02 |

*Note*. **p*<.05 ; ***p*<.01; SELF-T=self-transcendence, Unn=universalism-nature, Unc=universalism-concern, Unt=universalism-tolerance, Bed=benevolence-dependability, Bec=benevolence-caring, SELF-E=self-enhancement, Pod=power-dominance, Por=power-resources, OPEN=openness to change, Sdt=self-direction-thought, Sda=self-direction-action, CONS=conservation, Sep=security-personal, Ses=security-societal, Cor=conformity-rules, Coi=conformity-interpersonal, Work=physical activity in job-related domain, Transport=physical activity in transportation domain, House=physical activity in domestic domain, Leisure=physical activity in leisure-time domain, Global=total physical activity in four different domains.

**References**

Bilsky, W., Janik, M., & Schwartz, S. H. (2011). The Structural Organization of Human Values-Evidence from Three Rounds of the European Social Survey (ESS). *Journal of cross-cultural psychology*, 42(5), 759-776. http://doi.org/10.1177/0022022110362757

Borg, I., Groenen, P. J. F., & Mair, P. (2013). Applied Multidimensional Scaling. Berlin, Heidelberg: Springer-Verlag.

Chen, F. F. (2007). Sensitivity of goodness of fit indexes to lack of measurement invariance. Structural Equation Modeling, 14(3), 464-504.

Cieciuch, J., Davidov, E., Schmidt, P., & Algesheimer, R. (2016). Assessment of cross-cultural comparability. In C. Wolf, D. Joye, & T. W. Smith (Eds.), The SAGE handbook of survey methodology (pp. 628-646). Sage.·

Hu, L., & Bentler, P. M. (1999). Cutoff criteria for fit indexes in covariance structure analysis: Conventional criteria versus new alternatives. Structural equation modeling, 6(1), 1-55. http://doi.org/10.1080/10705519909540118

Lorenzo-Seva, U., & Ten Berge, J. M. F. (2006). Tucker's Congruence Coefficient as a Meaningful Index of Factor Similarity. Methodology, 2(2), 57-64. http://doi.org/10.1027/1614-2241.2.2.57

Schwartz, S. H. (1992). Universals in the Content and Structure of Values: Theoretical Advances and Empirical Tests in 20 Countries. Advances in experimental social psychology, 25(C), 1-65. http://doi.org/10.1016/S0065-2601(08)60281-6

Schwartz, S. H., Cieciuch, J., Vecchione, M., Davidov, E., Fischer, R., Beierlein, C., & King, L. (2012). Refining the Theory of Basic Individual Values. Journal of personality and social psychology, 103(4), 663-688. http://doi.org/10.1037/a0029393

Sturrock, K., & Rocha, J. (2000). A Multidimensional Scaling Stress Evaluation Table. Field methods, 12(1), 49-60. http://doi.org/10.1177/1525822X0001200104
